# Tracing nitrogen use efficiency of diverse Canadian spring wheat cultivars

**DOI:** 10.3389/fpls.2024.1439395

**Published:** 2024-12-02

**Authors:** Kate A. Congreves, Olivia Otchere, Pierre J. Hucl

**Affiliations:** Department of Plant Sciences, University of Saskatchewan, Saskatoon, SK, Canada

**Keywords:** nitrogen recovery, ^15^N labelling, fertilizer use efficiency, wheat breeding, spring wheat

## Abstract

Decades of wheat breeding have provided growers with numerous high-yielding options, but it is unknown if these yield improvements are likewise characterized with improved nitrogen use efficiency (NUE). Fertilizer nitrogen (N) is an ever-increasing expense, so improving NUE by reducing the requirement for N fertilizer without risking yield and quality is necessary. The goal of our research is to identify cultivars and associated traits that may improve NUE while maintaining productivity. We compared 25 spring wheat cultivars over a three-year period (2020, 2021, 2022) at two field sites differing in background soil N level for the ability to use fertilizer-N and allocate it to the grain. To do so, we employed the ^15^N stable isotope technique to trace the flow of fertilizer-N and determine the ^15^N recovery efficiency (^15^NRE). The ^15^NRE in the grain averaged 25.0% at the higher soil N site, and 15.5% at the lower soil N site. At the higher soil N site only, dwarfing alleles (Rht-B1b) were associated with greater ^15^NRE. Grain ^15^NRE was positively associated with yield, grain N content, and the ^15^N harvest index (^15^NHI) at the high soil N environment, but never at the low soil N environment. Our findings support the notion that the genetic development of high yielding semi-dwarf cultivars also translates into an improved ability to recover fertilizer-N—but this outcome is only expressed only under rich soil N conditions. Cultivars that simultaneously produced higher ^15^NRE and yields, grain N, or ^15^NHI differed by environment; possibly suggesting different mechanisms for improving crop NUE depending on background soil N level. Ultimately, cultivar-specific ^15^NRE information, including that presented here, will be useful breeders to design new crosses and approaches aimed at increasing NUE for spring wheat.

## Introduction

Wheat (*Triticum aestivum* L.) is instrumental to global food security. It is the most widely cultivated crop in the world due to its adaptability to diverse agroecological environments, storability, nutrient composition, and economic value (FAO, 2022a). Wheat supplies one fifth of total dietary calories and provides more protein than any other food source (FAO, 2022b, c). For such an important crop, inefficient production and failed harvests can have major geopolitical, socioeconomic, and environmental impacts. Consequently, wheat research and breeding efforts have developed cultivars with improved yields and end-use quality traits while also maintaining regional adaptation to stressors. Recent breakthroughs have delivered annotated reference genome sequences for bread wheat, laying the foundation for researchers and breeders to further advance wheat improvement (Appels et al., 2018). Despite this promising outlook for wheat improvement, there is one factor that has been elusive to improve but that has enormous impact on global food security—that being nitrogen use efficiency, NUE (Hitz et al., 2017).

Nitrogen (N), an essential nutrient for crops and often the most limiting soil nutrient, is regularly applied as a fertilizer to support yields. The problem is that our misuse, mismanagement, and modest understanding of NUE dynamics has had catastrophic consequences such that planetary boundaries have been exceeded for the N cycle (Steffen et al., 2015). Excess N resulting from agricultural production in some parts of the world has negative impacts on biodiversity, climate, and human health; yet, in other parts of the world, N shortages prevent food needs from being met (Stevens, 2019). In either case, improving NUE—the ability of plants to use the applied or available N—is key to the solution. Although the idea of breeding for increased NUE is not new and there is consensus that it is worthwhile goal, NUE is still not a commonly targeted strategy for breeding programs (Cormier et al., 2016). Instead, NUE has been influenced through indirect selection for yield or other traits. For example, the introduction of dwarfing genes (Rht) resulted in improved yield and harvest index (Gale and Youssefian, 1985; Ellis et al., 2002). Simultaneously, the dwarfing gene improved resistance to lodging, enabling crops to withstand greater rates of N fertilizer (Evans, 1997). However, with excessive N application wheat may only recover 40-45% of the N applied (Yu et al., 2022), typifying poor NUE and representing major inefficiency.

Nitrogen use efficiency is a complex trait with complex interactions, involving genetic and environmental factors and effects (Hawkesford, 2017). Nitrogen is taken up throughout the life cycle of wheat production; initially, N is taken up via seedling roots and helps support early crop establishment by supplementing seed N reserves. Nitrogen continues to be taken up as the plant develops, driving the establishment of the canopy and photosynthetic apparatus. If the environmental conditions are conducive, N continues to be taken up following anthesis and during grain filling. During grain filling, remobilization processes throughout canopy senescence move N from vegetative organs towards the grain. Identifying sources of variation in any of these processes are essential to breed improved NUE, but it is unclear how much variation exists for modern wheat cultivars and whether there is sufficient variation to improve NUE traits in future cultivars. To complicate the matter further, variation in grain yield is not a reliable proxy for improved NUE (Hawkesford and Griffiths, 2019). High rates of N fertilizer are usually applied in experiments where breeders are aiming to maximize genetic yield potential. However, without screening breeding lines in low N environments concurrently, it will not be possible to identify genotypes with robustly high NUE potential (Hitz et al., 2017). Significant improvements may be possible, both in N capture and in N conversion efficiency, but perhaps only through screening diverse germplasm material with substantial levels of trait variation (Hawkesford and Griffiths, 2019) and by considering its expression under a range of soil N environments (Hitz et al., 2017).

There are many ways to calculate NUE in crops (Congreves et al., 2021). The broad range of calculations means that the interpretation and applicability of NUE also varies widely, introducing uncertainty when determining crop NUE let alone trying to improve NUE. To better quantify crop NUE, more advanced techniques are needed. Stable isotopes of N (^14^N and ^15^N) can be used to better understand NUE because it enables researchers to track and distinguish N-derived from different pools (fertilizer versus indigenous soil N) into the plant and its distribution among plant organs. The vast majority (99.6337%) of N has an atomic mass of 14, whereas only 0.3663% has an atomic mass of 15 (Junk and Svec, 1958; Bedard-Haughn et al., 2003). As such, ^15^N has a natural abundance of 0.3663 atom% and is only slightly impacted by isotope discrimination during mineralization, immobilization, denitrification, and leaching (Robinson, 2001). Nitrogen-15 enrichment methods create differences in δ^15^N values via the addition of a known artificially enriched ^15^N source to the soil (i.e., fertilizer). Because the N source is enriched in ^15^N beyond the natural abundance levels, its subsequent processing, use, and flow can be traced and measured. In soil-plant research, this technique has been used to partition and trace the uptake of fertilizer N into variable soil and crop pools (Taveira et al., 2020; Farzadfar and Congreves, 2022). Without the use of ^15^N, it is not possible to differentiate the contributions of fertilizer-N to crop N use from other soil sources due to the large size, variability, and complexity of compounds that make up the soil N pool. Applying the ^15^N enrichment approach to identify variation in the N recovery efficiency among genotypes will enable researchers to quantify fertilizer recovery, rather than just estimate the apparent recovery. However, only a few studies have used this ^15^N technique to explore the genetic variation in NUE of wheat (Kumar et al., 2022, 2023; Paul et al., 2023). Determining the proportion of fertilizer-N that is allocated to grain is important for several stakeholders. It is a major factor in determining the cost benefit ratio of the economic crop component for growers; it plays a major role in developing wheat protein, a key quality metric and target for wheat breeders; it enumerates an N removal metric necessary for better modelling and understanding N balance budgets and N footprints. In contribution to addressing this gap, our objectives were to quantify NUE using the ^15^N tracer technique for a range of Canadian spring wheat cultivars (with and without Rht dwarfing genes) grown under low and high soil N environments, to explore the relationship between the ^15^N recovery efficiency (^15^NRE) in grain to other common traits (such as yield, grain N, and N harvest index, ^15^NHI), and ultimately to identify and inform breeding choices for improving spring wheat NUE.

## Materials and methods

We conducted a ^15^N tracer experiment to determine the N fertilizer use dynamics for 25 different spring wheat cultivars in 2020, 2021, and 2022. The experiment encompassed two sites, with different background soil N levels for a total of six site-years. Details of the ^15^N tracer approach and the agronomic experimental design are described below. Weather data was collected on a nearby climate station operated by Environment and Climate Change Canada.

The two sites were located on Dark Brown Chernozem soils with a clay loam texture near Saskatoon, Saskatchewan, Canada. The first site (52°10’25.000” N 106°43’08.001” W) was designated as the ‘high soil N environment’ due to average soil nitrate-N levels of 222 kg N ha^-1^ in 0-30 cm depth prior to planting. The second site 52°9’15.68” N 106°30’35.07” W was designated as the ‘low soil N environment’ due to average soil nitrate-N levels of 25 kg N ha^-1^ in the 0-30 cm depth prior to planting. Both sites have a history of grain crop production, previously growing barley, wheat, and/or pulse crops in rotation, managed with minimal soil disturbance (no-till seeding). The difference in background soil N is due to the legacy of fertilizer or manure applied in the past, informing the selection of each site. Soil characterization (including soil nitrate levels) were determined each spring prior to seeding by collecting several soil cores from randomly selected sampling points to gain a representative composite sample from across the entire plot area; soil samples were analyzed by A&L Laboratories, Ontario. At the high soil N environment, the soil (0-30 cm) contained an average 4% organic matter, pH of 7.3, cation exchange capacity of 30 m_eq_ 100 g^-1^, 45 mg kg^-1^ of extractable Bray-P, 712 mg kg^-1^ of extractable K, 18 mg kg^-1^ of extractable S. At the low soil N environment, the soil (0-30 cm) contained an average of 4% organic matter, pH of 7.5, cation exchange capacity of 31 m_eq_ 100 g^-1^, 13 mg kg^-1^ of extractable Bray-P, 380 mg kg^-1^ of extractable K, and 69 mg kg^-1^ of extractable S.

The experiment was arranged according to a randomized complete block design with four replications. Spring wheat cultivar was the treatment effect, where 25 different entries were grown, including Canadian western red spring (CWRS) class cultivars that do not carry a Rht-B1b allele for dwarfing, Marquis (designated as the main reference/control cultivar), AC Barrie, AC Elsa, and CDC Utmost; CWRS cultivars that are short and do carry the Rht-B1b allele include AAC Alida, AAC Brandon, AAC Connery, AAC Elie, AAC Starbuck, AAC Viewfield, AAC Wheatland, Carberry, Cardale, CDC Abound, CDC Go, CDC Hughes, CDC Landmark, Muchmore, and Stettler; other cultivars that also carry the Rht-B1b allele from the Canadian northern hard red (CNHR) class (Faller) and the Canadian prairie spring red (CPSR) class (AAC Goodwin); two near isogenic lines carrying the Rht18 gene (RHTNIL14012 and RHTNIL14029, derived from CWRS CDC Utmost and preliminary research indicated these show promise for grain N traits); for reference purposes, two short statured European cultivars from the Canadian western special purpose class were also included (Pasteur and Alderon). Note that Marquis is designated as the main reference/control cultivar due to its history and legacy on Canadian spring wheat, it is a result from the earliest breeding efforts to produce a spring wheat cultivar suitable for widespread production in western Canada, originally released in 1911 and remaining the dominant spring wheat cultivar in Canada until 1939 (McCallum and DePauw, 2008). Marquis became a crucial part of the quality standards for new cultivar registration as CWRS, and nearly all wheat cultivars bred over the past 100 years in Canada can be traced back to Marquis (McCallum and DePauw, 2008).

Each spring wheat cultivar was established in field plots of 1.2 by 3.7 m. Seeding took place on May 11, 8, and 15 in 2020, 2021, and 2022 respectively; seeds were drilled 3.8 cm deep with a planting density of 310 seeds m^2^ and row spacing of 20 cm. A starter fertilizer blend of 50 kg ha^-1^ of 28-23-0 was side-banded at seeding, at both sites. The consistent fertilizer applications at both sites ensured that fertilizer management was not a confounding variable, while also ensuring that the difference in background soil N levels between the two sites remained intact. Typical agronomic management practices including herbicide and pesticide applications were followed, as needed.

Once plants emerged and were at the three-leaf growth stage (between 220-260 growing degree days, GDD), microplots 0.5m^2^ (0.83 m x 0.61 m) were established with the main plots for the ^15^N experiment. The microplots were centered on the crop rows near the center of the main plots. Upon establishment, the microplots were delineated by a metal frame that was place on the surface of the soil. The microplots received ^15^N enriched fertilizer at a rate equivalent to 2 kg N ha^-1^ at 30 atom% ^15^N in the form of urea. The ^15^N labelled urea fertilizer was applied to the microplot by dissolving it in 1-2 L of water (depending on soil moisture conditions to allow for infiltration), and consistently applied to all plots by evenly distributing the solution across the surface of the microplot using a watering can. An additional 1-2 L of water was applied to ensure that the tracer moved into the soil. Water was applied slowly to avoid any lateral movement of applied urea solutions outside the frames.

Prior to anthesis between 525-550 GDD (July 6, June 29^th^, and July 4^th^ in 2020, 2021, and 2022), three random plants (one from each row within the microplots) were clipped at the soil surface and collected for biomass and ^15^N analysis. Plants were monitored throughout the growing season, recording date to head emergence, days to maturity, and plant height. At maturity between 1000-1200 GDD (August 25, 24, and 26^th^ in 2020, 2021, and 2022), plant samples were collected from each microplot by cutting all plants from the middle row (0.5 m transect) at the soil surface for biomass and ^15^N analysis. The mature samples were manually separated into different plant parts: lower leaves, flag leaf, stem, chaff, and grain. All samples were oven-dried at 60°C until constant weight to determine moisture fraction. Once all the ^15^N labelled samples were collected (including natural abundance controls from outside the microplots but within the main plots), wheat from the main plots were mechanically harvested, and grain was collected, and yields were recorded.

Plant sample preparation and analysis were conducted at the University of Saskatchewan. Grain and aboveground plant components were ground using a Wiley mill (Thomas Model 4, 800 rpm, using a 1 mm metal screen) after which a sub-sample of the milled plant material (~ 10 g) was ground to a powder using a Retsch ball grinder (Mixer Mill MM 200, shaking at 25 Hz for 2 min). The powdered samples were weighed (1-3 mg) into aluminum tin capsules to measure %N, %C, and bulk ^15^N abundances using gas chromatography-isotope ratio mass spectrometry (GC-IRMS – Thermo Scientific Delta V MS coupled with a Costech ECS4010 elemental analyzer; then switched to Elementar’s varioPYRO cube analyzer with a precision IRMS).

The recoveries of ^15^N labelled urea fertilizer in plant tissues (the different plant parts: lower leaves, flag leaf, stem, chaff, and grain) at harvest were calculated using the following equations:


$$NdfF=\;\frac{{\;}^{15}N\;atom\%\;excess\;in\;plant}{{\;}^{15}N\;atom\%\;excess\;of\;fertilizer\;N}\;\times \;100$$

$$TNdfF=\;\frac{Ndff}{100}\;\times \text{ Plant N}$$

$${\;}^{15}NRE=\frac{TNdff\;in\;Plant\;}{Fertilizer\;N\;applied}\;\times \;100$$

where the ^15^N atom% excess is calculated by subtracting the ^15^N atom% of natural abundance samples from that of the enriched samples; the NdfF is the proportion of N in the plant tissue that is derived from fertilizer; the TNdfF is the total amount fertilizer-N in the plant tissue; the ^15^NRE is the N recovery efficiency of the fertilizer-N applied that made it into the plant tissue. Further, the fraction of aboveground plant TNdfF (in all aboveground plant tissues: the lower leaves, flag leaf, stem, chaff, and grain) that was allocated to the grain was determined as ^15^NHI.

Statistical analysis was performed using GraphPad Prism version 9, GraphPad Software, San Diego, California, and alpha values were set at 0.1. Data from each soil N environment was analysed separately as our objective was to understand N dynamics from each scenario independently. For each site, data from all three years were pooled for a robust analysis of the cultivar effect, encompassing the interannual variability. Cultivar effects were explored using Kruskall-Wallis ANOVAs (replicates and years as random effects) followed by Dunn’s multiple means comparison tests (using the Marquis cultivar as the reference/control). Mann-Whitney rank sum tests were used to compare cultivars carrying the Rht-B1 gene to those without. Type II regression analyses were also performed to explore the linear relationship between grain ^15^NRE and other key variables (yield, grain N, and ^15^NHI). When significant regressions were identified but required some reassurance that the results were not unduly influenced by isolated characteristics of the data (i.e., a potentially high-leverage value representing the reference/control cultivar), the regressions were re-analyzed after deleting the value in question; in all cases, the original results were reassured (i.e., the regression p values remained significant, the direction/shape of the relationship remained consistent), warranting the inclusion of all values for analysis, data presentation, and interpretation.

## Results

### Growing season weather

**Figure 1:**
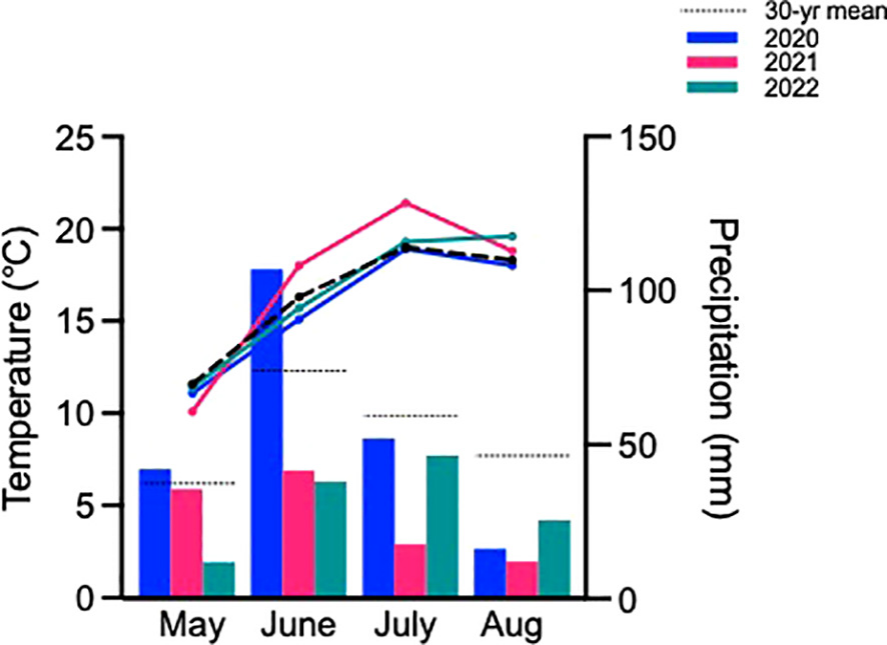
Monthly mean temperature and precipitation for the three growing seasons and both sites studied, as well as the 30-yr normal (1991-2020). Bars represent precipitation and lines represent temperature. Data from the Saskatchewan Research Council climate reference station and the Environment and Climate Change Canada reference station in Saskatoon.

Typically, growing seasons at this location experience average monthly temperatures of 16.3°C and accumulate 218 mm of precipitation based on 30-yr averages (**Figure 1**). Of the three growing seasons studied, 2020 was most representative of the norm with average monthly temperatures of 15.8°C and 217 mm of precipitation. In contrast, the 2021 growing season was warmer and much drier than usual especially as the season progressed, culminating with 17.1°C average monthly temperatures and only 107 mm of precipitation. The 2022 season was only marginally better than 2021, reaching 16.5°C average monthly temperatures and 122 mm of precipitation.

### Crop production metrics

**Figure 2:**
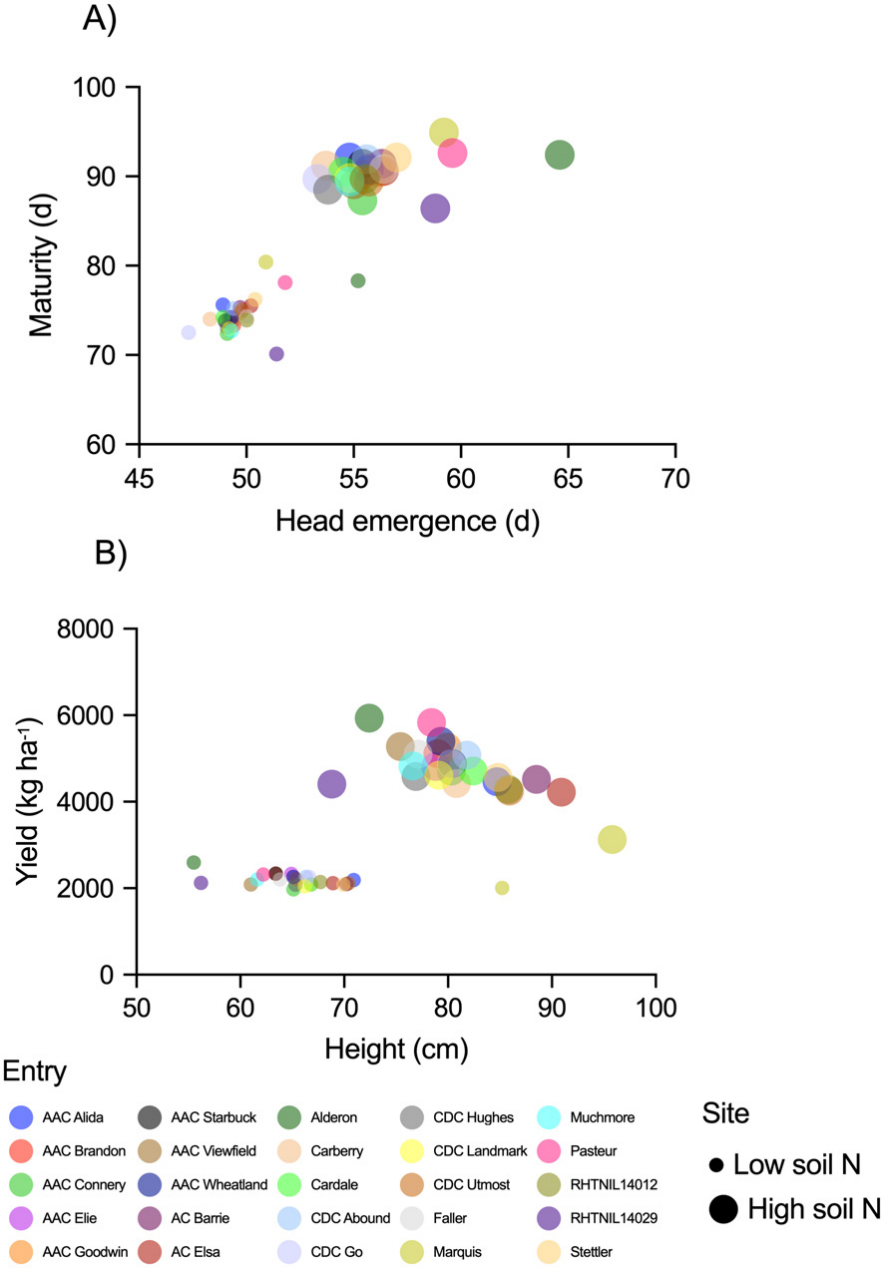
Spring wheat **(A)** average days to head emergence and maturity, and **(B)** average height and yields, for the three-year study period (2020, 2021, 2022). Markers represent average values (n=12) for each cultivar differentiated by color, and marker size differentiates the two sites (high *vs*. low soil N environments).

Average yield, height, days to head emergence, and days to maturity are shown in **Figure 2**. In general, wheat yielded 2 times more, grew 15 cm taller, reached head emergence 6 days later, and matured 16 days later when produced at the high soil N site than at the low soil N site. The relative difference in crop production metrics between the two sites was consistent across the three years, but 2021 (the exceptionally dry growing season) had the poorest metrics of all. At the high soil N environment average yields were 5108, 3900, and 5308 kg ha^-1^ in 2020, 2021, and 2022, respectively. The low soil N environment resulted in lower yields, averaging 2264, 1488, and 2767 kg ha^-1^ in the same three years, respectively. At either site, greater yields tended to be associated with shorter entries (**Figure 2**). Above-average yields *at both sites* were produced by AAC Brandon, AAC Elie, AAC Goodwin, AAC Starbuck, AAC Wheatland, Alderon, CDC Abound, CDC Go, Faller, Muchmore, and Pasteur.

### Nitrogen recovery efficiency

**Figure 3:**
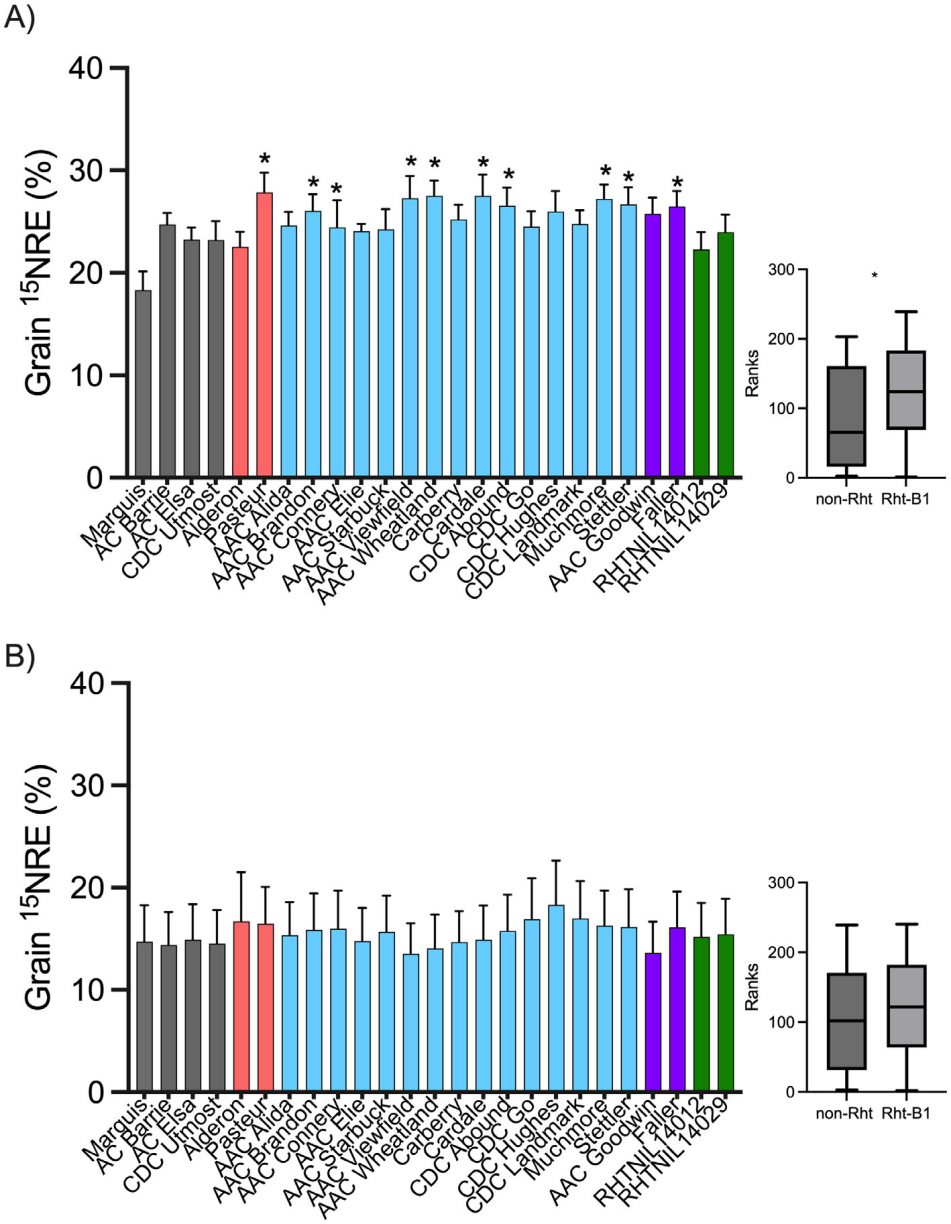
Fertilizer ^15^N recovery efficiency (^15^NRE) in the grain for spring wheat cultivars grown at the **(A)** high background soil N site and **(B)** low background soil N site, over the three-year study period (2020, 2021, and 2022). Bar graphs represents averages (n=12) ± standard errors. Colors indicate classification of cultivars as tall CWRS non-Rht-B1b (gray), European short (orange), short CWRS cultivars carrying Rht-B1b allele (blue), short CNHR and CPSR cultivars carrying the Rht-B1b allele (purple), and short NIL carrying the Rht-18 allele (green). At the high background soil N site, grain ^15^NRE varied by cultivar (Kruskall-Wallis p = 0.0614), and asterisks indicate significant differences (alpha< 0.1) relative to the Marquis reference according to a Dunn’s multiple means comparison test. At the low background soil N site, cultivars did not differ (Kruskall-Wallis p > 0.9999). Box-plots shows the Mann-Whitney rank test on ^15^NRE for Rht-B1b entries (blue) *vs.* non-Rht-B1b (gray) entries (asterisks indicates rank differences p< 0.001).

At the high soil N environment, the ^15^NRE in the grain averaged 25.0% with little variation across the three years—24.3, 23.5, and 27.3% in 2020, 2021, and 2022, respectively. Grain ^15^NRE varied by cultivar (p = 0.0614) where several cultivars had significantly greater ^15^NRE than the reference entry (Marquis), including CWRS Rht-B1 cultivars (AAC Brandon, AAC Connery, AAC Wheatland, Cardale, CDC Abound, Muchmore, and Stettler), CNHR Rht-B1 (Faller), and European cultivars (Pasteur) (**Figure 3**). At the high soil N site, greater ^15^NRE ranks and values were associated with short cultivars that carry the Rht-B1b allele compared to non-Rht-B1b cultivars (**Figure 3**).

At the low soil N environment, the ^15^NRE in the grain averaged 15.5% across all years, with similar recoveries in 2020 and 2022 (21.3 and 24.6%) but much lower values in the drought year (0.67%, almost no recovery of N in the grain in 2021), contributing to relatively higher variability in the average ^15^NRE results at this site (**Figure 3**). Regardless, there was no cultivar effect on grain ^15^NRE (p > 0.9999) and no differences in ^15^NRE relative to the reference cultivar, Marquis (**Figure 3**). Also at this site, there was no discernible difference in ^15^NRE ranks or values between the cultivars that carry the Rht-B1b allele compared to non-Rht-B1b cultivars (**Figure 3**).

Compared to the grain ^15^NRE, the whole plant ^15^NREs were 11.6 and 4.9% greater at the high and low soil N environments, respectively. Of the fertilizer N recovered by the plants, the majority (68 to 80%) was allocated to the grain. The plant stems recovered the second greatest proportion of fertilizer N (4.1 and 0.8%), followed by lower leaves (1.8 and 0.5%) or chaff (1.6 and 0.8%), and the flag leaf (0.6 and 0.1%) at the high and low soil N environments, respectively. Of the fertilizer N that was taken up by the plants throughout the growing season, 65 and 61% was acquired before anthesis at the high and low soil N environments, respectively.

### Predictors of fertilizer N recovery efficiency in grain

**Figure 4:**
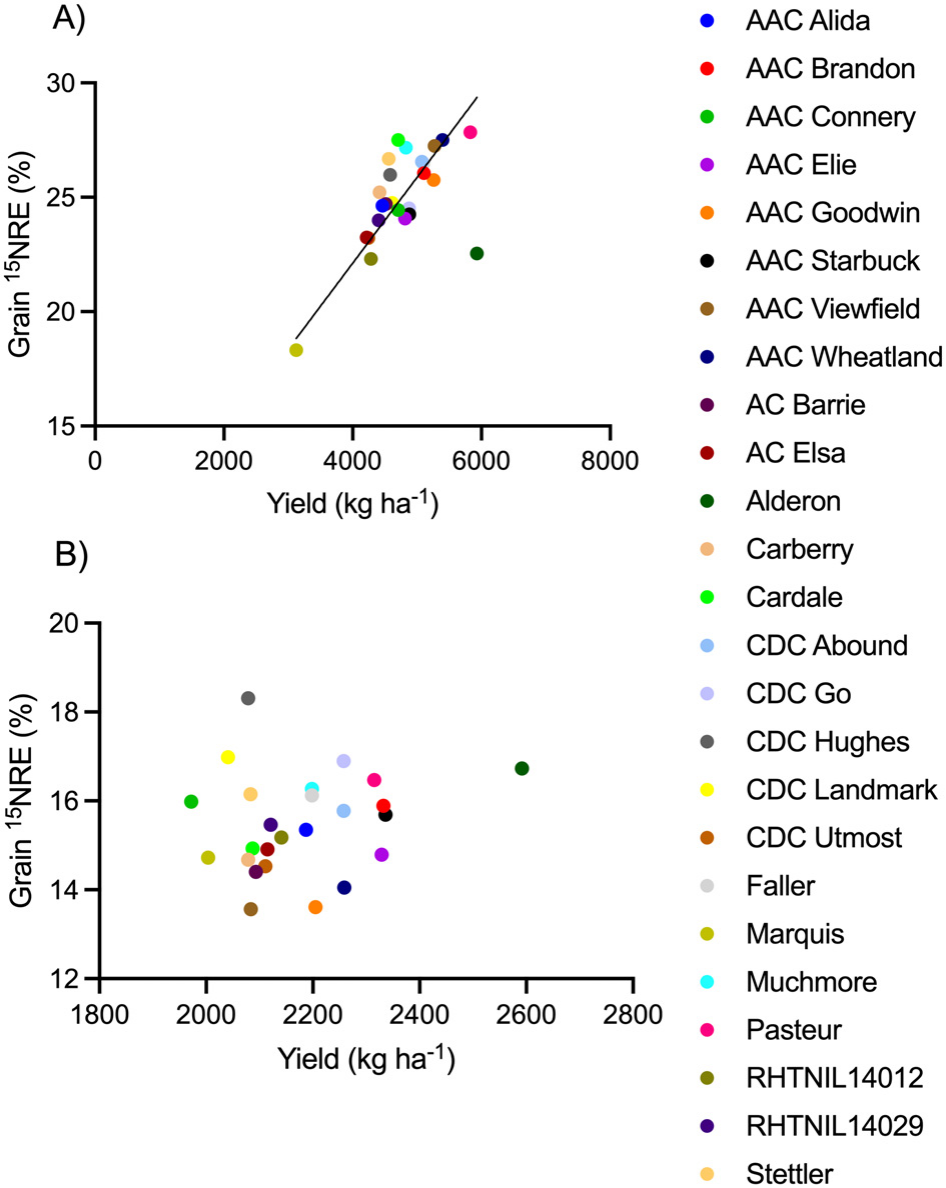
Relationship between grain fertilizer ^15^N recovery efficiency (^15^NRE) and yield at the **(A)** high soil N environment and **(B)** low soil N environment. Markers represent average values over the three-year study period (n=12) for each spring wheat variety, indicated with different colors. Where significant, linear regression with 90% confidence intervals shown as solid and dotted lines.

**Figure 5:**
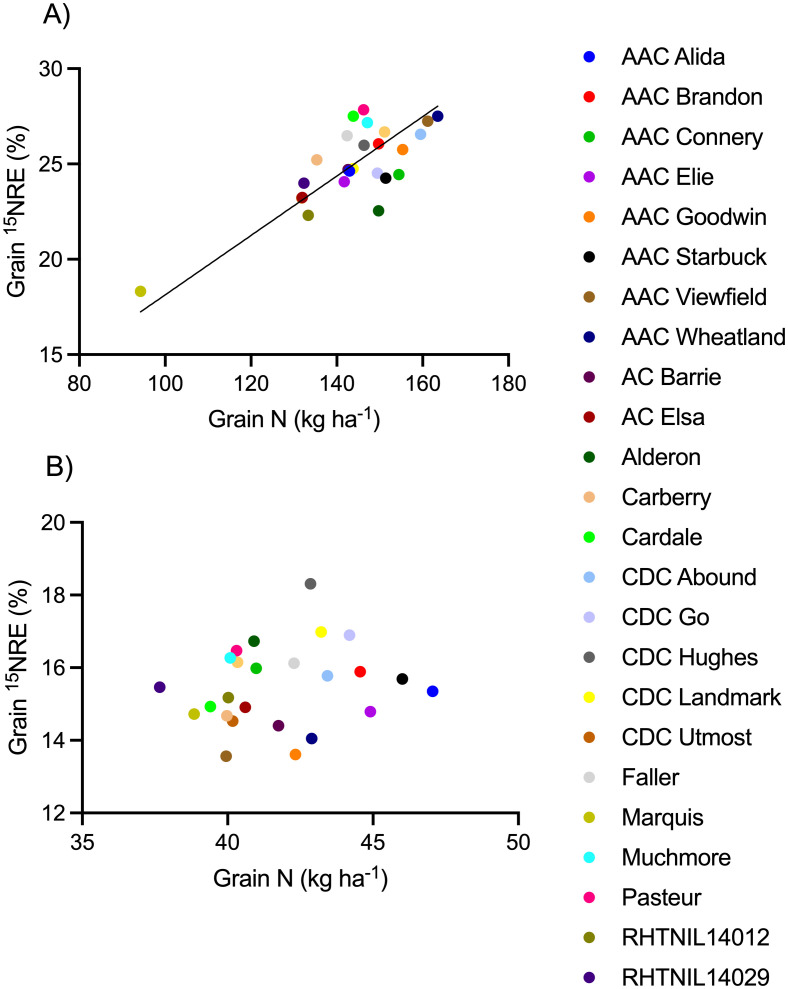
Relationship between grain fertilizer ^15^N recovery efficiency (^15^NRE) and grain N content at the **(A)** high soil N environment and **(B)** low soil N environment. Markers represent average values over the three-year study period (n=12) for each spring wheat variety, indicated with different colors. Where significant, linear regression with 90% confidence intervals shown as solid and dotted lines.

**Figure 6:**
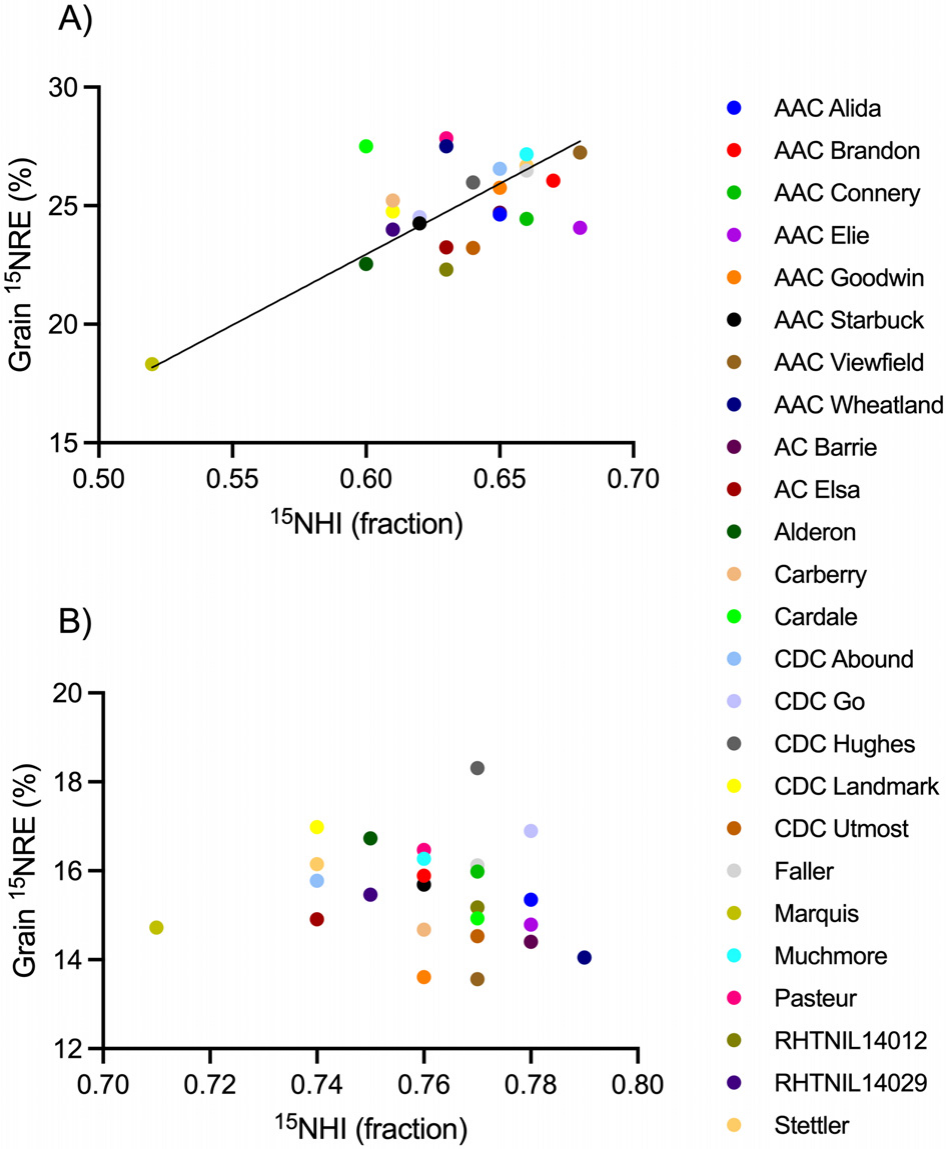
Relationship between grain fertilizer ^15^N recovery efficiency (^15^NRE) and the ^15^N harvest index (^15^NHI) at the **(A)** high soil N environment and **(B)** low soil N environment. Markers represent average values over the three-year study period (n=12) for each spring wheat variety, indicated with different colors. Where significant, linear regression with 90% confidence intervals shown as solid and dotted lines.

Spring wheat yield (**Figure 4**), grain N content (**Figure 5**), and ^15^NHI (**Figure 6**) were significant predictors of grain ^15^NRE at the high soil N environment (p< 0.001, p< 0.001, and p = 0.001, respectively) but not at the low soil N environment (p = 0.407, p = 0.359, and p = 0.531, respectively. Unlike the high soil N environment where all relationships were significant and positive, there was no clear association between the variables at the low soil N environment.

The cultivars that simultaneously produced the greatest (top 10%) yield *and* ^15^NRE were, at the high soil N environment: Pasteur and AAC Wheatland (**Figure 4**). But, at the low soil N environment, different cultivars topped the list, Alderon and CDC Go (**Figure 4**). In either case, it appears that one European cultivar and one CWRS cultivar carrying the Rht-B1 gene were top performers. Cultivars that performed the poorest (bottom 10%) for yield *and* ^15^NRE were Marquis at both soil N environments, plus RHTNIL14012 at the high soil N environment and AAC Viewfield at the low soil N environment.

Cultivars that simultaneously produced the greatest (top 10%) grain N content *and* ^15^NRE were, AAC Viewfield and AAC Wheatland at the high soil N environment, but CDC Go and CDC Hughes at the low soil N environment (**Figure 5**). All four are CWRS cultivars that carry the Rht-B1b gene. Cultivars that performed the poorest (bottom 10%) for grain N *and* NRE were the same ones as identified for yield *and* NRE, above.

Selecting for the greatest performance for ^15^NHI *and* ^15^NRE concurrently, AAC Viewfield and Muchmore were among the top 10% of the dataset at the high soil N environment, and CDC Go and CDC Hughes were greatest at low soil N environment (**Figure 6**). All four are CWRS cultivars that carry the Rht-B1b gene. Cultivars that performed the poorest (bottom 10%) for ^15^NHI *and* ^15^NRE were Marquis at both soil N environments.

### Relationship of production metrics when grown at high versus low soil N environments

**Figure 7:**
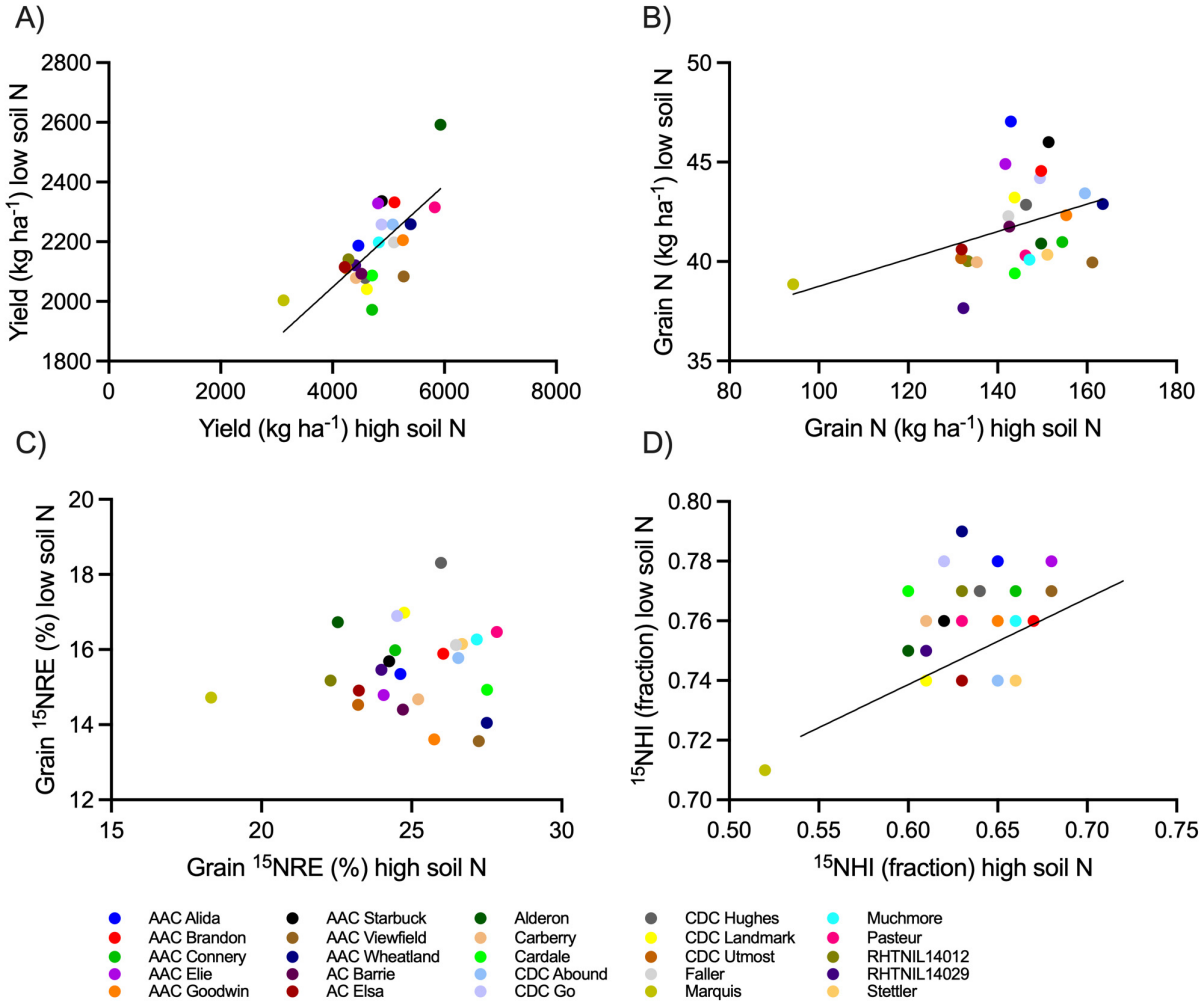
Relationship of spring wheat production metrics when grown at the high soil N environment relative to the low soil N environment. Metrics include **(A)** yield, **(B)** grain N, **(C)** grain 15NRE, and **(D)** 15NHI. Markers represent average values over the three-year study period (n=12) for each spring wheat variety, indicated with different colors. When significant at alpha < 0.05, linear regressions are shown (solid lines) with 90% confidence intervals (dotted lines).

For the low soil N environment, spring wheat yield, grain N, and ^15^NHI were significantly predicted from the high soil N environment results, but not ^15^NRE (**Figure 7**).

## Discussion

Despite exhaustive research focused on crop NUE and related traits (Muurinen et al., 2007; Garnett et al., 2015), our understanding of the genetic basis for these traits remains highly uncertain. Nitrogen use efficiency is complex involving genetic, environmental, and management factors and interactions that fluctuate with time, space, plant species, growth, and nutrient acquisition strategies. Using traditional approaches that rely on determining total N concentrations and plant N contents, it is very difficult to achieve accuracy and precision in determining NUE, let alone appropriately conceptualizing NUE. Nonetheless, researchers persist in studying NUE with the goal of better understanding the genetic basis of NUE so that we can design innovative breeding strategies for developing improved NUE crop cultivars. Our research sought to elucidate how variation in wheat genotypes can potentially shape NUE. The approach using ^15^N provided unique insight on the recovery of fertilizer N by wheat plants that is impossible via traditional methods without the use of stable isotopes. Additionally, we sought to evaluate the role of background soil N levels on the recovery of fertilizer in the grain, and the relationship of N recovery to other key agronomic traits under divergent soil N conditions.

Grain ^15^NRE varied by cultivar at the high soil N environment where greater ^15^NRE was associated with short cultivars carrying the Rht-B1b allele, but no differences were observed at the low soil N environment (**Figure 3**). It is possible that short cultivars carrying the Rht-B1b alleles have a propensity to improve grain ^15^NRE but only when grown under higher soil N environments. This makes sense because semi-dwarf wheat cultivars (commonly carrying a Rht gene) typically have greater resistance to lodging due to shortened stems and increased straw strength (Ellis et al., 2002) thereby enabling plants to better withstand richer N environments that would otherwise induce excessive N uptake and result in lodging (Evans, 1997). Similarly, long strawed cultivars of wheat, barley, and oat have been characterized by low N uptake efficiency (Muurinen et al., 2007). Under lower soil N environments where there is little chance of excessive plant N uptake, the advantage of reduced lodging might not translate into an improved N recovery effect. Our findings suggest that when searching for genetic basis for NUE (conceptualized here as fertilizer N recovery in the grain), semi-dwarf cultivars may provide clues only when grown under high soil N environments. Whereas yield and grain N content grown under the high soil N environment reasonably paralleled those from the low soil N environment, grain ^15^NRE was not (**Figure 7**). This finding adds further evidence that genetic expression of improved NUE depends on background soil N, unlike other traits like yield or grain N (a proxy for protein). Perhaps the higher soil N environment led to a shallower more branched root system that was better able to capture the ^15^N fertilizer applied, providing more inference space for different cultivars to fully develop and express differences in grain ^15^NRE.

Screening genotypes under low soil N environments may reveal different mechanisms for improving crop NUE than indicated by high N environments. While one might expect higher ^15^NRE under lower background soil N conditions, if low background N conditions are combined with another stressor (like the dry conditions experienced in our study), poor crop performance may be exacerbated, thereby impairing ^15^NRE. Interestingly, grain ^15^NRE was positively associated with yield, grain N content, and ^15^NHI only at the high soil N environment, never at the low soil N environment in our study. Further, certain cultivars performed dramatically differently depending on the soil N environment. For instance, AAC Viewfield was a top performer in terms of grain ^15^NRE and N content at the high soil N environment, but it was among the poorest performers for grain ^15^NRE and yield or N when grown at the low soil N environment. Similarly, when evaluating the relationship between the timing of N uptake and winter wheat production, Hitz et al. (2017) found yields were more highly correlated to post-anthesis N uptake under high soil N environments but only minimally so under low soil N environments. These findings indicate that the processes of N translocation and remobilization to the grain may play a larger role in explaining NUE under higher soil N environments. Does this mean that other processes (perhaps root N transport, acquisition, and/or scavenging) play a more dominant role in explaining NUE under lower soil N environments? In soil science research, wheat genotypes were found to differ in root C allocation patterns, influencing N cycling (Kelley et al., 2022). These researchers found that genotypes with thicker roots released more C into soil, which enhanced N mineralization through stimulation of the microbial biomass; microbial biomass then increased N-cycling enzyme activity and soil N-uptake by wheat. As such, genotypes with the propensity to influence soil N cycling processes via key root traits might translate into improved NUE—most applicable to low soil N environments. Better understanding how to improve ^15^NRE when crops are grown under low soil N environments (and combined with abiotic stressors like drought) will be useful for advancing crop production in regions and scenarios where soil N supply is limiting.

Breeding choices for developing improved NUE might be derived from genotypes that performed well in both soil N environments. In terms of agronomic performance, we must consider grain ^15^NRE alongside other metrics like yield, N content, or NHI. In our study, yield, grain N content, and ^15^NHI were positively related to ^15^NRE at the high soil N environment only. Genotypes that tended to produce high yields, grain N, or NHI at the same time as producing high ^15^NRE differed by environment. At the high soil N environment, AAC Wheatland and AAC Viewfield topped the list in two of the three assessments. At the low soil N environment, different cultivars prevailed, CDC Go in all three assessments, and CDC Hughes in two of the three assessments. These genotypes should be further explored in the pursuit of better understanding the genetic basis of NUE and developing breeding strategies for improved NUE. In doing so, care must be taken to ensure that the performance is applicable to a wide range of soil N environments, and not just to high soil N backgrounds.

Without using a ^15^N tracer, NUE can be conceptualized as constituting two independent traits, namely, N uptake efficiency (NUpE) and utilization efficiency (NUtE)—and researchers have tried to determine which trait has a greater influence on cereal crop NUE (Beatty et al., 2010; Fiaz et al., 2021). In evaluating spring wheat cultivars, researchers found that NUpE was more strongly correlated with NUE than NUtE (Muurinen et al., 2006), although the two independent traits (NUpE and NUtE) are often negatively correlated with each other (Hawkesford and Riche, 2020). Recently, a global meta-analysis showed a significant non-linear relationship between wheat yield and NUpE (de Oliveira Silva et al., 2020), further supporting the notion that improving N uptake is key to developing breeding strategies for improved NUE. To achieve this, it is crucial to consider the timing of N uptake and focus on N taken up early in the growing season. About 70% or more of yield N is a result of N remobilized from vegetative tissues during senescence (Muurinen et al., 2007)—a statistic confirmed by our ^15^N study, where 68-70% of the fertilizer N recovered by the plants was translocated to the grain. Hence, focusing on traits that support early N uptake will likely translate into improved NUE. Our research offers a foundation for subsequent genetic studies, for example, the use of quantitative trait loci (QTLs) mapping methods to discover genes of significance for NUE, as done for barley and producing lines with 20-40% higher NUE (Garnett et al., 2015). Through the recent advancement in genome studies, several QTLs controlling NUE were identified i.e., 15 QTLs in barley, 4 in rice and several in wheat (Fiaz et al., 2021). Other work with Arabidopsis thaliana and rice recommends investigating and modifying key genes responsible for N metabolism to improve NUE in wheat (Islam et al., 2021).

As previously discussed, certain cultivars performed differently depending on the soil N environment. Contrastingly, such variability was not observed in other experiments, although in these examples, ^15^N tracers were not used. Namely, spring barley (var. Vivar) showed similar NUE when grown on either a high or low N soil, despite yield penalties at the low N site (Beatty et al., 2010). Further, N remobilization efficiency of wheat, barley, and oat was generally greater under higher than lower soil N conditions (with wheat producing the lowest values), but there was no two-way interaction between cultivar and soil N level (Muurinen et al., 2007). In another study that compared spring barley genotypes grown under different N levels in field vs growth chamber conditions, NUE was similar regardless of the environment (Beatty et al., 2010). Differing background N levels can influence key development phases in which N plays an important role. For example, higher soil N availability may delay leaf senescence, in effect creating longer photosynthetic periods that supply more photosynthates to the grain. This extended period also reduces N remobilization rates, risking lower grain N content in some cases (Fiaz et al., 2021), but possibly increasing grain NUE throughout the entire growing season due to the longer period of time for remobilization. Differences in the interplay of these mechanisms (time, translocation, uptake, remobilization, senescence) may explain differences in grain ^15^NRE between our sites, but another key variable is how this network of relationships might be regulated by genetics—and more challenging to decipher. Recently, Hawkesford and Riche (2020) observed differences in N utilization efficiency among 15 wheat cultivars, irrespective of soil N levels, and attributed this variation to their respective inherent yield potentials. The uptake of nitrogen compounds by plant roots are regulated by transporters distinct from absorption to translocation within the plants. Under elevated or low N concentrations, the low-affinity and high-affinity transport system (LATS and HATS) employ either the nitrate or ammonium transporter in the uptake of N from the soil. In evaluating rice, the ammonium transporter was associated with improving NUE compared to its counterpart, the nitrate transporter (Fiaz et al., 2021). Other traits such as leaf characteristics play a vital role in N partitioning throughout the plant; and the variations at temporal and spatial scales which control N cycling also influence the amount of N allocation (Bedard-Haughn et al., 2003).

## Conclusions

Nitrogen use efficiency is a complex trait, but tracing fertilizer ^15^N into crops and their components is a powerful approach to better understanding N flow, allocation, and use. The goal of this research was to identify genotypes and traits associated with improved NUE by focusing on a selection of 25 Canadian spring wheat cultivars and employing the ^15^N tracer technique to quantify ^15^NRE. Grain ^15^NRE averaged 25.0% at the higher soil N site, and 15.5% at the lower soil N site. At the higher soil N site only, dwarfing alleles (Rht-B1b) were associated with greater ^15^NRE. This finding supports the notion that the genetic development of semi-dwarf cultivars (intended to reduce lodging issues) also translates into an improved ability to recover fertilizer-N—but this outcome is only expressed only under rich soil N conditions. In exploring relationship between ^15^NRE and other traits, grain ^15^NRE was positively associated with yield, grain N content, and ^15^NHI only at the high soil N environment, never at the low soil N environment in our study. The cultivars that simultaneously produced the greatest (top 10%) yield *and* ^15^NRE were, at the high soil N environment, Pasteur and AAC Wheatland. But, at the low soil N environment, different cultivars topped the list, Aldernon and CDC Go. Screening genotypes under low soil N environments may reveal different mechanisms for improving crop NUE than indicated by high N environments. Ultimately, ^15^NRE information will be useful for breeders to design new crosses and approaches aimed at increasing NUE for spring wheat.

## Data Availability

The raw data supporting the conclusions of this article will be made available by the authors, without undue reservation.
